# The evolution of strongly-held group identities through agent-based cooperation

**DOI:** 10.1038/s41598-021-91333-w

**Published:** 2021-06-08

**Authors:** Roger M. Whitaker, Gualtiero B. Colombo, Yarrow Dunham

**Affiliations:** 1 School of Computer Science and Informatics, Cardiff University, 5 The Parade, Roath, Cardiff, CF24 3AA UK; 2 Crime and Security Research Institute, Cardiff University, Friary House, Greyfriars Rd, Cardiff, CF10 3AE UK; 3 Department of Psychology, Yale University, Box 208205, New Haven, CT 06520 USA

**Keywords:** Human behaviour, Psychology, Mathematics and computing, Coevolution, Cultural evolution, Social evolution

## Abstract

Identity fusion represents a strongly-held personal identity that significantly overlaps with that of a group, and is the current best explanation as to why individuals become empowered to act with extreme self-sacrifice for a group of non-kin. This is widely seen and documented, yet how identity fusion is promoted by evolution is not well-understood, being seemingly counter to the selfish pursuit of survival. In this paper we extend agent-based modelling to explore how and why identity fusion can establish itself in an unrelated population with no previous shared experiences. Using indirect reciprocity to provide a framework for agent interaction, we enable agents to express their identity fusion towards a group, and observe the effects of potential behaviours that are incentivised by a heightened fusion level. These build on the social psychology literature and involve heightened sensitivity of fused individuals to perceived hypocritical group support from others. We find that simple self-referential judgement and ignorance of perceived hypocrites is sufficient to promote identity fusion and this is easily triggered by a sub-group of the population. Interestingly the self-referential judgement that we impose is an individual-level behaviour with no direct collective benefit shared by the population. The study provides clues, beyond qualitative and observational studies, as to how hypocrisy may have established itself to reinforce the collective benefit of a fused group identity. It also provides an alternative perspective on the controversial proposition of group selection - showing how fluidity between an individual’s reputation and that of a group may function and influence selection as a consequence of identity fusion.

## Introduction

The role of strongly-held identities in motivating human behaviour, particularly in respect of groups, has gained widespread interest^1^. In particular, identity fusion^2^ has made significant progress in characterising the causal mechanisms that lead individuals to act with extremist pro-group actions that appear self-less. Unlike preceding theories on social identity (e.g.,^3,4^), identity fusion postulates that individuals retain a personal identity that has a porous boundary with a group identity, meaning that the personal and group identity become integrated, rather than a dominant single identity fluctuating (i.e., group verses personal identity). Critically, through identity fusion, individuals do not become depersonalised, and instead, both individual and group identities persist, with characteristics of group identity being evident in the individual’s sense of self. Consequently when identity fusion is strong, behaviours that occur in support of one’s identity are also pro-group. The implications of this mechanism are significant since a group can benefit from the acts of an individual while that individual remains self-focused. This may promote positive survival benefits for a group, but it can also support extremism, through so-called “devoted actors”^5–11^, defined as individuals fused with a group, with which they share a sacred value. These individuals become fully aligned with all-consuming group beliefs that motivate extreme behaviour, to the extent that actors disassociate themselves from any consideration of personal risk and costly sacrifice. Furthermore, identity fusion predicts not only extreme self-sacrifice with a group, but also for an ideology, another individual, or even other entities^1^.

Described as a visceral feeling of oneness with the group^2^, identity fusion has been developed from extensive empirical observations that bring together principles of personal agency^12^, identity synergy^13,14^, the saliency of relational ties^15^ and irrevocability^16^, resulting in measures of identity fusion^12^ that assess the permeability of an individual’s personal and social self, including the development of a verbal scale^17^. This has enabled wide-ranging insights to be established, helping to explain diverse pro-group behaviours such as loyalty^5,18^, altruism^19^, self-sacrifice^20^, extreme cooperation^21^ and nationalism^22^.

In contrast to these empirical breakthroughs, relatively little attention has addressed the evolutionary origins of strongly-held group identities such as identity fusion^9,21^. This can be explored through an experimental approach using agent-based modelling, which is a long standing, valuable and potentially underused method in social psychology^23^. In particular, agent-based modelling has predominantly been used to model the fast evolution of minimal groups (e.g.,^24–28^), where elegantly simple assumptions on agent-based interaction result in remarkable group-level coordination. However, agent-based modelling has had limited application in exploring strongly-held identities^21^, which are inherently more complex to model because of the necessary recourse to underlying qualitative theory^1^. In this paper we present a first approach to exploring *how and why might identity fusion establish itself in an unrelated population with no previous shared experiences*. The conditions under which self-sacrifice becomes incentivised has received greater attention with existing literature providing useful clues in support of the hypothesis that pro-group sacrifices may be a by-product of one or more basic survival mechanisms^2^. This builds on inclusive fitness^29,30^ as a means for individuals to promote the survival of one’s genes through support for those with whom they are shared, with evidence of correlation between self-sacrificial behaviour and genetic relatedness across a wide range of species^31–35^. However inclusive fitness alone is insufficient to explain how such behaviour may become embedded in unrelated individuals. Research on human kin detection^36^ further indicates that small group interactions may have co-evolved as a proxy for genetic relatedness, for example through perinatal associations, which implicitly support inclusive fitness by promoting the groups in which they evolve.

However, identity fusion is prevalent beyond this context, in large unrelated groups rather than small tribal groups, where individuals do not a-priori interact (i.e., so-called extended fusion). Swann et al^2^ comment that a form of “shared essence”^37^ may have emerged where biological behaviours have transferred to the social world, contributing to the visceral feeling that binds an individual to the group, based on observations of natural human tendencies to conceptualise the world based on biological survival mechanisms (e.g.,^38–41^). These observations point to the important role that evolution has likely played in explaining identity fusion, however the possible mechanisms that are responsible for its promotion are open to further consideration^2^. In particular, *beyond kinship, have particular mechanisms evolved that allow identity fusion to be incentivised and establish itself?*

Agent based modelling requires a framework for interaction between agents, and we explore identity fusion using indirect reciprocity^42–46^. This is a fundamental form of ‘one-shot’ interaction and across all species only humans fully engage with indirect reciprocity^47,48^, which is thought to have established itself through the management and assessment of an agent’s reputation^49^. As a first model for the evolution of strongly-held identities across a population with no a-priori relationships or shared experiences, we adopt indirect reciprocity for two main reasons. Firstly, it presents an opportunity to extend a simplistic approach to modelling identity, since the widespread convention for indirect reciprocity is to assume that all agents have a unique identity with no component being influenced or shared with others^42–44,50–52^. Secondly, significant progress has been made based on this assumption, providing a baseline for comparison^53^. We make progress noting that the composition of an agent’s identity determines how its reputation is derived and subsequently used by others in decision-making for potential one-shot cooperation. This approach is aligned to the particular context of indirect reciprocity where reputation is an important characteristic. However, we note that alternative attributes beyond an agent’s source of reputation could be used to facilitate identity.

**Table 1:** Key parameters of the model for an agent *i*.

Parameter(s)	Description	Role in model	Subject to natural selection
$u_i, d_i, s_i$	Rules for *i*’s donation based on self-comparison of potential recipient *j*’s reputation $r^j$ with $r^i$	Governs *i*’s donation behaviour	Yes
$f_i$	Identity fusion level for agent *i*	The extent to which *i* derives its personal identity from group *G*	Yes
$r^j$	Donor i’s view of agent j’s reputation. When $f_i=0$, $r^j = r_j$. When $f_i >0$, $r^j = (1-f_j)r_j + f_jr_G$.	Reputation incorporating j’s representation of identity and the extent of its fusion with group *G*	No
$r_i$	Personal reputation for an agent *i*	Personal identity for agent *i* which accrues as a consequence of *i*’s actions	No
$r_G$	Group reputation	Group identity that is acquired by agents through their identity fusion towards *G*	No
$ctr_i$	An additional personal reputation for fused agents capturing their support for group *G*	Basis for a fused agent to detect hypocrisy, given the fusion level of another agent	No
$n_i^{pos},n_i^{neu},n_i^{neg}$	Number of donation actions taken by agent *i* that respectively: contribute to group *G*’s reputation; leave group *G*’s reputation unchanged; reduce group *G*’s reputation	Definition of contribution level $ctr_i$	No
$S_i$	Probability that i plays the donation game with a randomly chosen member of the in-group. $S_i$ fixed exogenously or $S_i = f_i$	Governs how well-mixed the interactions are between agents in different groups	No
*p*	Probability that a fused agent *i* checks for hypocrisy in the potential recipient *j*	Allows fusion to correlate with vicarious hypocrisy, with $p = f_i$	No
*T*	Minimum threshold on an agent’s fusion level before consideration of ostracism is invoked	Allows assessment of whether just highly fused agents can invoke fusion in the wider population	No

**Table 2:** Interpretation of the model’s key parameters.

Parameter(s)	Theoretical/empirical justification
$r^j$	Reputation $r^j$ for an agent *j* indicates how others may perceive *j*’s overall identity taking into account *j*’s fusion with the group *G*. Individual reputations have been widely accepted as providing an explanation for different forms of collective action^49^, based on agent identities being independent (i.e., mutually exclusive). To generalise this we let $r^j$ incorporate identity fusion^2^, involving an individual’s isolated personal identity overlapping with that of a group through its fusion level $(f_j)$. This was originally conceived for human assessment of identity fusion^54^, and is based on the subject intersecting circles representing their personal identity and the group’s identity. The extent of intersection of these circles has been found to strongly correlate with the subject’s identity fusion $f_j$^17^, leaving a proportion of $1-f_j$ of *j*’s personal identity that is excluded from overlap with group *G*’s identity on the Venn diagram. We use this representation as a first proxy to represent an integrated identity for *j*, based on reputations aligned with personal identity $(r_j)$ and group identity $(r_G)$, to derive *j*’s integrated reputation $r^j$, where $r^j = (1-f_j) r_j + f_j r_G$. Conservatively, we assume this occurs only when the donor is itself fused (i.e., $f_i > 0$) and therefore can place value in the group’s reputation. Otherwise *i* perceives *j*’s reputation $r^j$ as $r^j = r_j.$ This ensures that when *i* is unfused $(f_i =0)$ its behaviour defaults to a base model^53^ for indirect reciprocity involving only individual isolated reputations. This guards against inadvertently promoting fusion during the agent interaction stage based on implicit assumptions of agent awareness of group *G*
$s_i, u_i, d_i$	These binary variables define the current strategy for agent *i*’s decision-making in the donation game, and are based on the social comparison of the potential recipient’s reputation with that of the donor, *i*. Without consideration of identity fusion, the strategy of donating to those with a similar or greater reputation is known to evolve and sustain cooperation (i.e., $s_i = u_i = 1, d_i = 0$)^53^. Originating from Festinger^55,56^, it is evident that self-referential evaluation frequently influences decision making from a social perspective^57–59^. Social comparison is also phylogenetically ancient^60^ and embedded in evaluating competitors and assessing whether or not to commit resources in wide ranging contexts^61–68^. These variables are subject to evolution and coevolve with an agent’s identity fusion $f_i$.
$S_i$	The in-group for an agent *i*, is defined as those with at least the same level of identity fusion as *i*, or greater. The in-group is introduced to accommodate possible effects concerning homophily^28,69^, allowing an agent to preference interaction with others that have a common social identification (i.e., at least the same level of identity fusion). $S_i$ controls the probability of *i* playing the donation game with a randomly selected partner *j* from the in-group during step (1) of the model, as opposed to *j* being randomly selected from the whole population with probability $1-S_i$. Homophily is known to establish itself through evolutionary means^25,28,70,71^ and here we control $S_i$ exogenously, using a range of values including $S_i = 0$. While we are not aware of research establishing that fusion heightens the probability of in-group interaction, we accommodate this possibility by also considering the hypothetical case that $S_i = f_i$.
$ctr_i$	This represents an additional form of personal reputation for fused agents, noting that under identity fusion, personal identity remains salient for fused agents. This assesses the relative contribution (proportion of actions from a generation’s start) that an agent is making in support of group *G*, thus reflecting their in-group behaviour. It enables fused agents, who are assumed to have heightened sensitivity to pro-group behaviours, to observe the contribution of other fused agents. This leaves agents open to experiencing inconsistencies, relative to themselves, concerning support for *G*. Through a ‘visceral sense of oneness’^21,54,72,73^ with *G*, we assume a highly fused agent *i* may experience vicarious hypocrisy^74^ when a higher fused agent *j* is observed contributing less support to *G* than *i* (i.e., $ctr_i > ctr_j$). We assume this invokes cognitive dissonance^75–77^ for *i*, which is received through a group connection with *j* due to fusion with *G*^78,79^. In response, agent *i* is assumed to invoke a form of ostracism^2,80^, because this is the lowest cost mitigation^81^ that an agent can make to reduce its dissonance. This aligns with a heightened disposition to maximise in-group advantage^82^ despite the additional overhead to agent *i.*
*p*	*p* controls the probability that an agent checks the extent that another agent’s contribution towards *G* (i.e., agent *i* compares $ctr_i$ against a given agent *j*’s contribution $ctr_j$) is justifiable, again based on self-referential social comparison^55,56^. We assume $p = f_i$, which aligns with an agent having a heightened incentive to consider the pro-group behaviour of other agents when its own fusion level is greater^82^. This assumption results in an increased chance of checking as fusion increases and reflects an increasing pro-group motivation due to an agent’s increased oneness with the group *G*^21,54,72,73^. This also ensures that the personal identity of fused agents remains salient as their fusion increases.
*T*	Checking the contribution of other agents incurs an overhead for the agent involved and is more relevant to those with greater dependency on fusion towards the group for their own identity. *T* defines a threshold when agent interest in checking the contribution of others is triggered, and enables the exploration of highly fused sub-groups, which are important to consider^5–9.^

Our agent based model involves six steps as indicated below, based on the parameters presented in Table 1. Steps $(1)-(4)$ represent an agent-based model of cooperation, with the respective components being well-studied in their own right and (mostly) not derived from identity fusion theory. Identity fusion comes into play mainly through step 1 (agents expressing the extent to which they are fused with the group identity), in step 5 (representing a further proxy for personal reputation, allowing in-group assessment between fused agents) and step 6 (behavioural responses for fused agents in light of hypocrisy).

Step (1) - *Identity and reputation.* Each agent *j* expresses the extent of its identity fusion $f_j$ towards a group *G*, where $0 \le f_j \le 1$, and $f_j$ is subject to evolution. We follow the identity fusion assessment approach^54^ developed for human subjects to express an overlap between their personal identity and a group identity on a pictorial basis through a Venn diagram. The extent that a human subject intersects a circle representing their personal identity with a circle representing the group *G*’s identity has been shown to strongly correlate with the individual’s level of identity fusion $f_j$ towards *G*^17^. This approach presents a region of *j*’s personal identity circle that does not overlap with the group *G*, representing $1-f_j$ of *j*’s personal identity circle. Through this model, a fused human participant *j* expresses $1-f_j$ of their personal identity as being independent of *G*, and $f_j$ of their identity as intersecting with *G*’s identity. We adopt this simple conceptual representation, as used to assess human subjects^17,54^, as a first proxy for an agent’s reputation. That is, when $f_i>0$ then we assume agent *i* views *j*’s integrated reputation as approximated by:

1 $$\begin{aligned} r^j = (1-f_j) r_j + f_j r_G \end{aligned}$$

where $r_j$ is the *personal reputation* that is unique to *j* and a consequence of its actions, while $r_G$ represents the *group reputation* which is shared with those having non-zero fusion. When $f_i=0$ then *i* views *j*’s reputation $r^j$ independent from *j*’s identity fusion towards *G*, irrespective of *j*’s fusion level, specifically:

2 $$\begin{aligned} r^j = r_j \end{aligned}$$

Equation (2) represents a conservative assumption with respect to identity fusion, ensuring that when *i* is unfused (i.e., $f_i = 0$), interactions default to a base model^53^ involving only individual isolated reputations. It also aligns with *i* not needing to value *G*’s reputation when it is not a member (we note that if Equation (2) is removed and all agents adopt Eq. (1), similar results are obtained, but with fusion evolving slightly more strongly in particular scenarios).

When viewed in isolation, Eq. (1) appears to diminish the importance of agent *j*’s personal reputation as *j*’s fusion $f_j$ increases. However, it is important to note that Eq. (1) does not function in isolation. Specifically, as the observing agent *i*’s fusion level increases, a further measure of agent *j*’s personal standing relative to *G* is increasingly applied (see step (5) and $ctr_j$). Here $ctr_j$ represents a personal reputation measure of *j*’s standing with respect to the group, ensuring that *j*’s personal identity remains salient even when *j* is highly fused. In other words, *j*’s personal identity is not substituted for the group identity as fusion increases, which is defining characteristic of identity fusion.

Step (2) - *Agent interaction.* Pairs of agents, say *i* and *j*, are repeatedly selected at random to play the donation game, a generalisation of the mutual aid game^83^, where agents choose whether or not to donate at cost *c* to a recipient who gains benefit $b> c > 0$, without any guarantee of future reciprocation. If *i* decides to donate to *j* then *i* incurs a cost *c* while the recipient *j* receives a greater benefit *b*, where $b> c > 0$. Accrued benefits and costs result in an agent’s payoff. *i*’s donation decision is based on simple self-comparison^53^ of its reputation $r^i$ with $r^j$. Action rules held by each agent, denoted by the binary vector $(s_i,u_i,d_i)$, govern whether or not donations take place, based on similarity ($s_i$), upward comparison ($u_i$) or downward comparison ($d_i$) of *j*’s reputation $r^j$ relative to $r^i$. $(s_i,u_i,d_i)$ represents *i*’s donation strategy which is subject to evolution, with (1, 1, 0) known to dominate and sustain cooperation in the absence of identity fusion^53^.

It is often the case that homophily can influence interactions. This is widely seen in nature^27^ and can invoke in-group effects^71^. Here we model this using variable $S_i$ (distinct from $s_i$) which governs the probability that agent *i* randomly selects an interaction partner *j* from its so-called in-group, which is defined as the subset of agents having at least the same level of fusion as *i* (i.e., at least $f_i$ in common). Alternatively agent *i* randomly selects *j* from the whole population of agents with probability $1-S_i$. This models fusion as a point for homophilic attraction and provides an in-group aligned to common pro-group identities^12^. We consider both the effects of exogenous fixed values for $S_i$, as well as considering personal fusion supporting a hypothetical pro-group disposition to interact with fused others (e.g., $S_i = f_i$).

Step (3) - *Updating reputation.* The donation decision that *i* makes in respect of *j* potentially results in an update to the sources of *i*’s reputation (possibly $r_i$, $r_G$ or both) based on *assessment rules*. These represent the social norms^84–86^ that govern whether donation behaviour should be rewarded or penalised, and reflect a form of morality^87^. While many alternative assessment rules have been considered^42,88–92^, we apply standing^88,93,94^ which avoids penalising agent *i* for a lack of donation to those viewed as more limited cooperators. We use integer steps between -5 and +5 for reputations $r_i$ and $r_G$. Specifically, if *i* donates to *j* then both $r_i$ and $r_G$ are incremented. If *i* defects on *j* and *j* is at least as reputable as *i* ($r^j \ge r^i$) then both $r_i$ and $r_G$ are decremented. If *i* defects on *j* and *j* is less reputable than *i* ($r^j < r^i$) both $r_i$ and $r_G$ remain unchanged. This assumes that *i* is fused ( $f_i > 0$) and therefore *i*’s reputation is dependent on both $r_i$ and $r_G$. When $f_i = 0$ the above updating rules are only applied to $r_i$.

Step (4) - *Selection and reproduction.* After *m* interactions between agents, the end of a single generation is reached and selection is modelled. Payoff, as a consequence of making and receiving donations gives a basis for fitness (see step (2)), where agents update their future strategy based on copying the action rules and fusion level of another agent with probability proportional to its relative fitness across the population. This roulette wheel approach follows the long standing basis for asexual reproduction^95^ and represents a form of social learning commonly applied in the evolution of indirect reciprocity^42,44,96,97^. Payoff and all reputation variables are set to zero in preparation for the next generation to commence.

Step (5) - *Personal identity remains salient for fused agents.* Under identity fusion, it is not to be expected that strongly fused individuals solely derive their identity and reputation from the group’s identity, and personal identity remains highly salient. Because fused individuals see themselves and other group members through an individualising lens, they take into account their personal standing and that of other group members when they consider each other’s positioning. Therefore we assume fused agents undertake further assessment between themselves based on their personal contribution ($ctr_i$) towards the group. This represents an additional form of personal reputation for fused agents specifically aligned to their in-group behaviour.

Note that fused agents have a personal incentive to support pro-group behaviour and the group’s competitive advantage^82^ due to their identity being drawn in part from the group. There are many ways in which awareness of perceived inconsistent behaviour of other group members could present itself to an agent and invoke a response through extended fusion^2,72^. Based on the fundamental role of social comparison^55,56^ in group processes, which enables group members to navigate and understand their positioning and social context^98,99^, we assume a simple (self-referential) approach, based on detecting inconsistency in others personal behaviour as compared to oneself. This aligns with experiencing vicarious hypocrisy^74,100^. It models the potential for a fused agent to experience cognitive dissonance^76,78,79^ due to the perceived hypocritical personal identity^77^ of agents who are connected through co-dependency on fusion with *G*.

We use an additional form of personal reputation ($ctr_i$) through which fused agents can experience potential hypocritical behaviour with their peers. Within a generation, this considers the number of times a fused individual *i* ($f_i > 0$) takes actions (i.e., makes donations) that contribute to group *G*’s reputation ($n_i^{pos}$) or actions (i.e., legitimate defections) that leave group *G*’s reputation unchanged ($n_i^{neu}$) as a proportion of the total actions that affect group *G*’s reputation $r_G$. Defining $n_i^{neg}$ as the number of actions made by *i* in reducing *G*’s reputation ($r_G$) then we define an agent’s *group contribution ratio*, $ctr_{i}$ as:

3 $$\begin{aligned} ctr_{i} = \frac{n_i^{pos} + n_i^{neu}}{n_i^{pos} + n_i^{neu} + n_i^{neg}}. \end{aligned}$$

This additional form of personal reputation for fused agents gives a basis for agent *i* to consider whether another fused agent *j* is hypocritical in the group context. We assume this occurs when *j* exhibits the same or greater level of fusion as *i*, but a comparably lower level of contribution to sustaining group *G*’s reputation, specifically:

4 $$\begin{aligned} f_j \ge f_i \text{ and } ctr_j < \ ctr_i. \end{aligned}$$

We assume that the motivation to consider perceived hypocrisy positively correlates with an agent’s identity fusion level, since the perception of an in-group deviate is strengthened^101^ when the agent’s personal role in the identity of that group is high. Therefore an agent *i* applies hypocrisy detection (Equation (4)) on a potential donor *j* with probability *p*, where $p=f_i$.

Step (6) - *Responding to hypocrites.* Behavioural responses to manage cognitive dissonance can take many potential forms^79,102,103^. Here we apply the most basic response, involving the exclusion of others, or *ostracism*^2,80^. This effectively provides a “cost-less punishment”^81^ and its positive effects in sustaining cooperation have been well-established^51,104–106^. Ostracism can be applied at the interaction stage (step 2) which we call *type-1*, or at the reproduction stage (step 4) which we call *type-2*, or both stages, denoted *type-3*. Under type-1 ostracism at the interaction step, *j* does not receive a donation from *i*, irrespective of *i*’s action rules. Under type-2 ostracism at the reproduction step, then *j* becomes excluded as a candidate to replace *i* in the next generation. These responses correspond to individuals discounting those that are a threat to their own identity due to this being fused with that of the group. To understand the sensitivity of fusion upon ostracism, we also invoke a minimum threshold *T* ($0< T < 1$) that an agent’s fusion level must reach ($f_i \ge T$) for any ostracism (type-1, type-2 or type-3) to take place. This enables exploration of highly fused sub-groups.

The modelling assumptions in these six steps incorporates possible relevant behaviours that align with drivers from identity fusion (see Table 2), and it is possible that many alternatives could be studied. We also note that this agent-based modelling approach deviates from traditional methods used in social identity research and highlights wider opportunities to study the evolution of individual differences in group attachment. A summary of the key parameters is presented in Table 1. Further implementation details are provided in the Methods Section.

Using the six modelling steps, we experiment with the key parameters and features ($S_i$, ostracism, *T*, execution and perception errors, and cost benefit ratio *c*/*b*) to understand the conditions under which identity fusion ($f_i$) is potentially established, promoted and sustained across the population of agents who interact through indirect reciprocity. All results are the average of five runs, and we apply $m=5000$ rounds of the donation game per generation, over 50,000 generations, using a *c*/*b* ratio of 0.7. Additionally, at the start of each generation, mutation is performed on each element of an agent’s action rule at a rate of 1%, and the new fusion level at a rate of 1%. These settings are based on previous exploration of the parameter space^53,107^ combined with additional consideration of the fusion parameter. Throughout we apply a population of 100 agents. This commonly used population size^24–27,42,44,53,70,83,107,108^ provides sufficient scale to observe group-based phenomena while also being computationally tractable.

## Results

### Ostracism in response to vicarious hypocrisy supports the coevolution of identity fusion and cooperation

**Figure 1:**
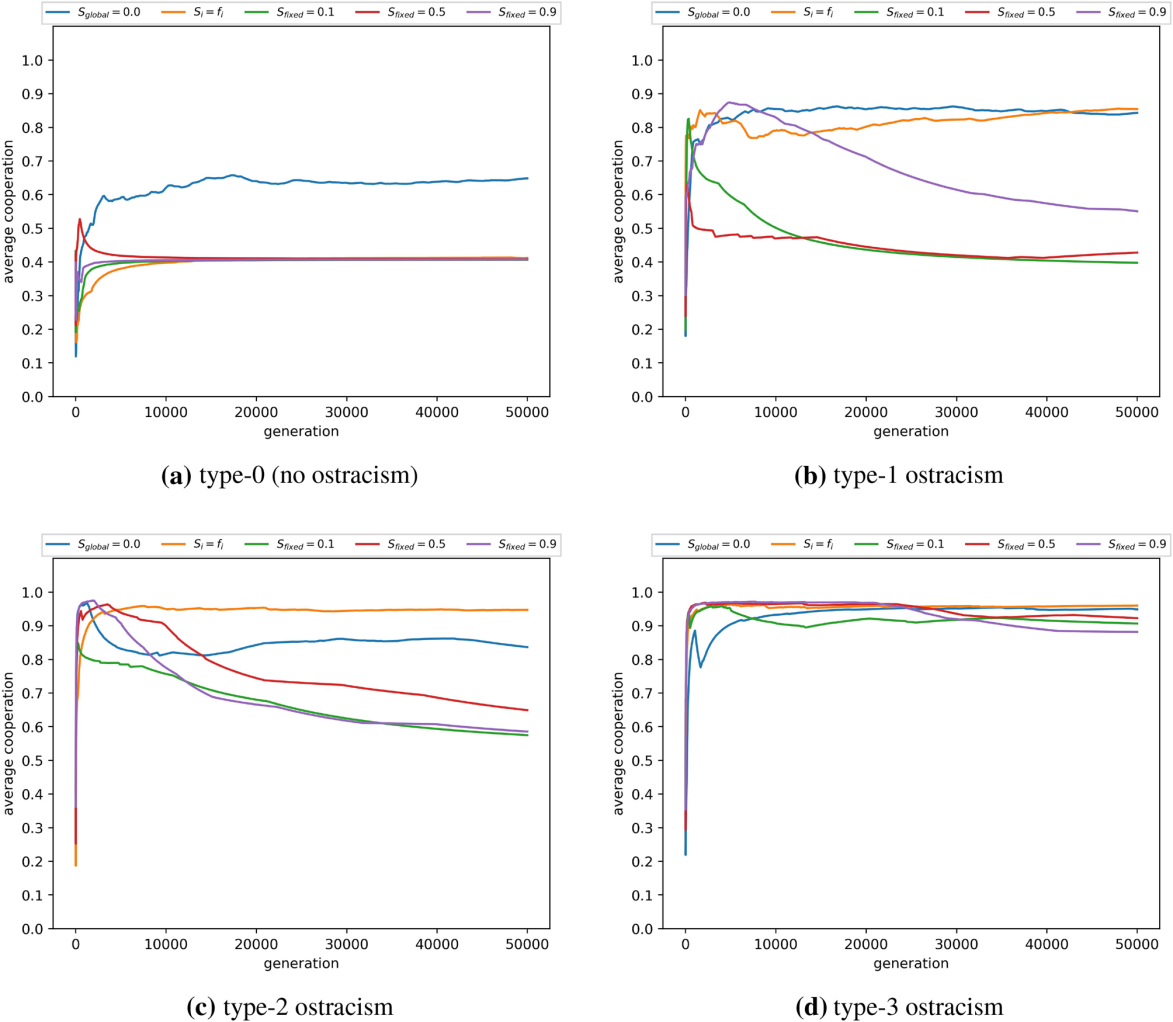
Average cooperation per generation for type 1 (**b**), 2 (**c**) or 3 (**d**) ostracism. Type 0 (**a**) represents a scenario without ostracism. Ostracism is performed by each agent *i* with a probability equal to the agent’s fusion level $f_i$. Average cooperation indicates the cumulative frequency of cooperative interactions, defined as the total number of donations made in all preceding generations as a proportion of the total number of games played in all preceding generations. Agents interact randomly with the other fused individuals within their in-group with fixed probabilities of $S_i$ ($S_i = 0.1, 0.5, 0.9$), or with a probability equal to their own current fusion level ($S_i = f_i$). $S_{global}$ indicates that all agents mix uniform randomly (i.e., $S_i = 0$). The in-group of an agent *i* is defined as the subset of agents having at least same level of fusion as *i* (i.e., $f_i$ or greater). Agents interact randomly with the whole population with a probability equal to $1 - S_i$. Results are averaged over five randomly seeded runs. Number of agents $N=100$; number of games per generation $m=5000$; number of generations $M=50,000$. Action rules and fusion levels are mutated at the rate of 0.01 per generation.

**Figure 2:**
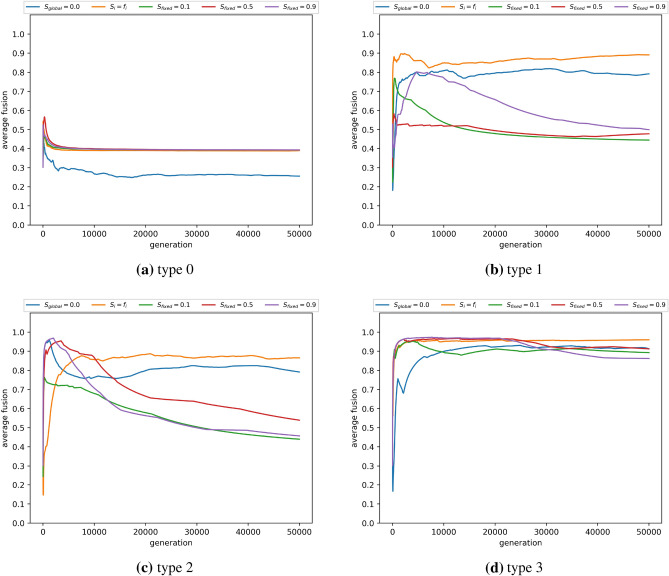
Average fusion per generation for type 1 (**b**), 2 (**c**) or 3 (**d**) ostracism. Type 0 (**a**) represents a scenario without ostracism. Ostracism is performed by each agent *i* with a probability equal to the agent’s fusion level $f_i$. Average fusion is the sum of all occurrences of fusion levels seen up to and including the current generation, divided by the number of generation-agent pairs (the number of generation-agent pairs is the current generation number multiplied by the number of agents). Agents interact randomly with the other fused individuals within their in-group with fixed probabilities of $S_i$ ($S_i = 0.1, 0.5, 0.9$), or with a probability equal to their own current fusion level ($S_i = f_i$). $S_{global}$ indicates that all agents mix uniform randomly (i.e., $S_i = 0$). The in-group of an agent *i* is defined as the subset of agents having at least same level of fusion as *i* (i.e., $f_i$ or greater). Agents interact randomly with the whole population with a probability equal to $1 - S_i$. Results are averaged over five randomly seeded runs. Parameter settings are consistent with Fig. 1.

We assume that $T=0$, indicating that all fused agents undertake either type-1, type-2 or type-3 ostracism. The baseline for comparison is the condition where no exclusions are made (i.e., type-0 ostracism). Here, it is known that when identity is represented by individual isolated reputation (i.e., $f_i = 0$, $\forall i$), then cooperation readily emerges^53^. Fusion disrupts this by providing opportunities for shirkers (i.e., agents with the defective strategy) to gain payoff while using the shared group reputation. The average result for the baseline scenario (Figs. 1 and 2) mask a cyclical phenomena where cooperation and fusion co-evolve until shirkers infiltrate the population, and both cooperation and fusion collapse.

Under type-1 ostracism, exclusion from donation impedes the payoff for individuals with strategies that are potentially inconsistent in supporting group reputation $r_G$. In contrast, type-2 ostracism functions without disruption to the donation game, but restricts the propagation of candidate strategies from fused agents that are deemed detrimental to sustaining the reputation of *G*. This means that type-1, type-2 and type-3 ostracism provide a defence against exploitation of shared group identity, albeit with different levels of effectiveness, dependent on the conditions for in-group interaction ($S_i$). Figure 1 shows that $S_i$ has considerably different effects on average cooperation, depending on the form of ostracism that is applied. Without ostracism (type-0, Fig. 1a), externally fixing $S_i$, or assuming $S_i$ aligns with fusion level (i.e., $S_i = f_i$) equally caps average cooperation to low levels, and fusion levels are negligible (type-0, Fig. 2a) as they present a mechanism for defective strategies to take hold.

However type-1 and type-2 ostracism (Fig. 1b,c) reflect that when the higher fused are increasingly more likely to interact in-group (i.e., $S_i = f_i$), then islands of cooperation form within the highly fused groups. Type-1 ostracism introduces discrimination at the interaction stage that dis-incentivises defective behaviour towards fused agents. This allows donations to be received from highly fused individuals and in turn incentivises fusion (Fig. 2b,c), creating a dependency where cooperation and fusion can coevolve. When $S_i = f_i$, payoff from cooperative actions of fused individuals are more likely to remain between fused individuals, meaning that selection, when combined with type-2 ostracism, impedes fused individuals from adopting strategies that conflict with their dependency on the group identity. This provides an advantage to fused agents who receive payoff benefits from the cooperative and fused. Note that this dynamic does not establish itself when the probability of in-group interaction is fixed rather than being correlated with fusion (i.e., $S_i = f_i$).

### Responding to hypocrisy at both interaction and reproduction reduces sensitivity to the extent of in-group interactions

**Figure 3:**
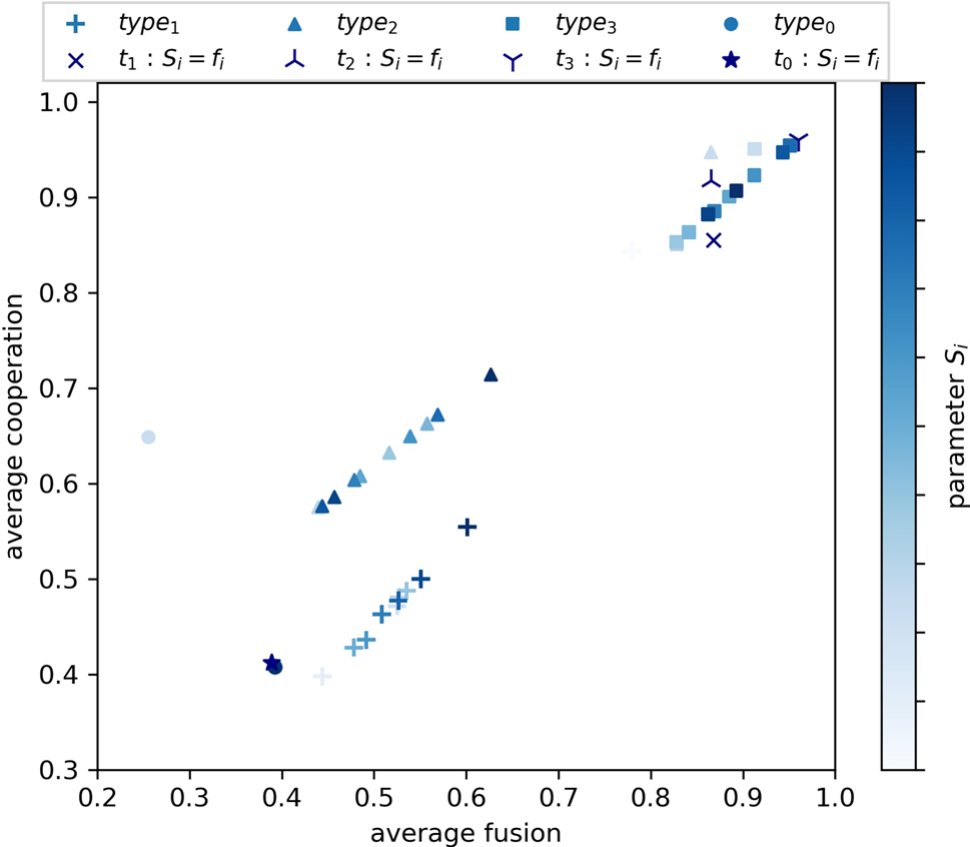
Average cooperation as a function of the average level of fusion for type 0, 1, 2 and 3 ostracism, denoted *t*_*0*_, *t*_*1*_, *t*_*2*_ and *t*_*3*_ respectively. Ostracism is performed by each agent *i* with a probability equal to the agent’s fusion level $f_i$. Each data point is referenced by different probabilities of in-group interactions $S_i$, which indicates the probability that an agent *i* interacts with a randomly chosen member of the in-group. The in-group of an agent *i* is defined as the subset of agents having at least same level of fusion as *i* (i.e., $f_i$ or greater). $S_i$ is defined as either a fixed probability within the range [0,1] or correlated with the agent’s fusion level (e.g., $S_{i}=f_i$). Agents interact randomly with the whole population with a probability equal to 1 - $S_i$. Results are averaged over five randomly seeded runs, each conducted for 50,000 generations. Parameter settings are consistent with Fig. 1.

Interestingly, when type-1 and type-2 are combined there appears to be a compound effect occurring (type-3, Figs. 1d, 2d), that is sufficient to promote cooperation when the population mixing structure is exogenously fixed throughout, across a wide span of $S_i$ values. While type-1 and type-2 ostracism show significant variation in response to in-group interaction, this is not the case for type-3, with average cooperation and average fusion both achieving greater than 85%. In contrast high sensitivity to the structure of interactions (i.e., the extent of in-group interactions) affects type-1 and type-2 ostracism, for both average cooperation and average fusion (Figs. 1b,c, 2b,c). These sensitivities can be seen in Fig. 3, where the spread of performance in both dimensions is an approximate linear function of in-group mixing ($S_i$). The results affirm that the action of ostracism, motivated by hypocrisy, is a highly effective strategy for incentivising a shared group identity that aligns with cooperation. However this needs to take place at multiple points in an agent’s activity (i.e., both interaction and reproduction) to be robust to in-group mixing when this is exogenously controlled by the scenario (i.e., $S_i$ fixed).

### The response of only the highly fused individuals to hypocrisy is sufficient to invoke population-wide fusion

**Figure 4:**
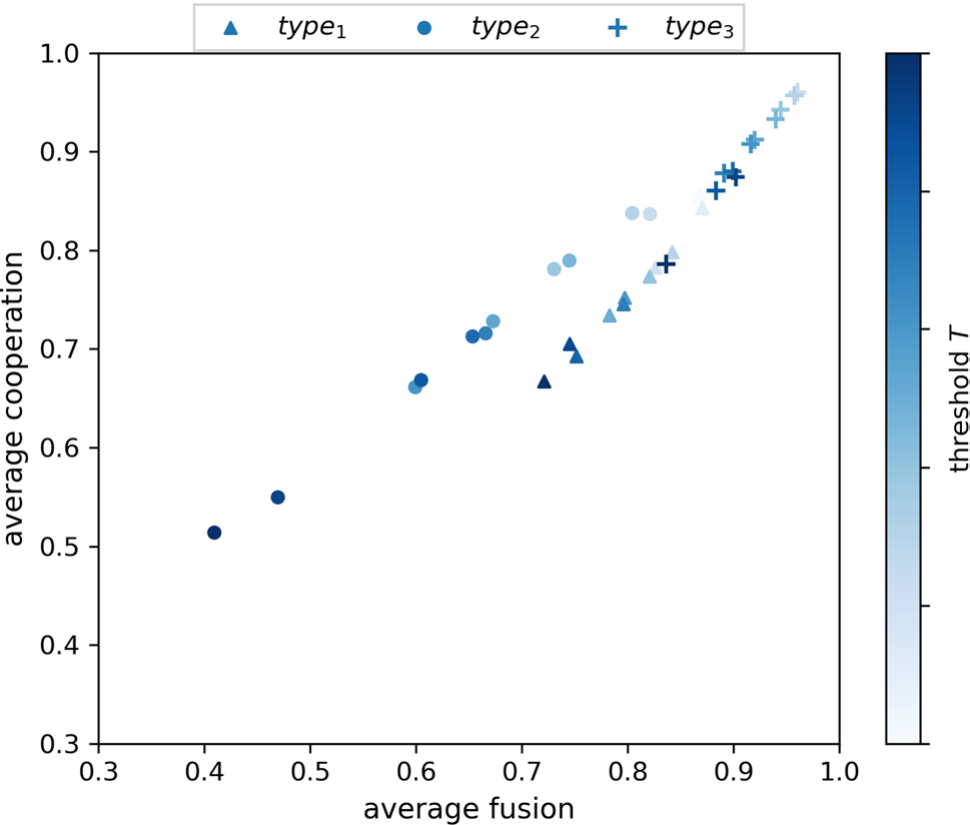
Average cooperation as a function of the average level of fusion for type 1, 2 and 3 ostracism. Ostracism is performed by each agent *i* with a probability equal to the agent’s fusion level $f_i$. Each data point is referenced by different probabilities of a threshold $T$ defined as the minimum threshold that an agent’s fusion must reach in order for ostracism to take place ($f_i \ge T$). $T$ is defined as a threshold value within the range [0,1] fixed for each of the agents. Agents interact randomly with the other fused individuals within their in-group with a probability equal to their own fusion levels ($S_i = f_i$). The in-group of an agent *i* is defined as the subset of agents having at least same level of fusion as *i* (i.e., $f_i$ or greater). Agents interact randomly with the whole population with a probability equal to 1 − $S_i$. Results are averaged over five randomly seeded runs, each conducted for 50,000 generations. Parameter settings are consistent with Fig. 1.

Figure 4 shows that if ostracism is invoked by only the most highly fused, then it is sufficient for both cooperation and fusion to coevolve. This indicates that those with the greatest fusion levels can act, through discrimination towards hypocrisy, to protect the group reputation on which they are highly dependent. Collectively this provides fused agents with an advantage where fusion becomes aligned with payoff, leading to promotion and escalation of the fused population. These observations assume that agents interact more frequently with other fused individuals based on their own fusion levels ($S_i = f_i$), and the results apply strongly for type-3 ostracism, but less so for type-2 ostracism, and not for type-1 ostracism in isolation.

These results also emphasise that only a subpopulation needs to be compliant in terms of adherence to detection and response to hypocrisy, and this sub-population aligns with those agents that naturally have the greatest motivation to act in this way due to the nature of identity fusion. Note that additionally, the behaviour of the subgroup results in the wider population being incentivised to diminish the isolated (i.e., non-group) element of personal reputation, through fusion towards the group.

### Coevolution of fusion and cooperation is robust to perception and execution errors on hypocrisy

**Figure 5:**
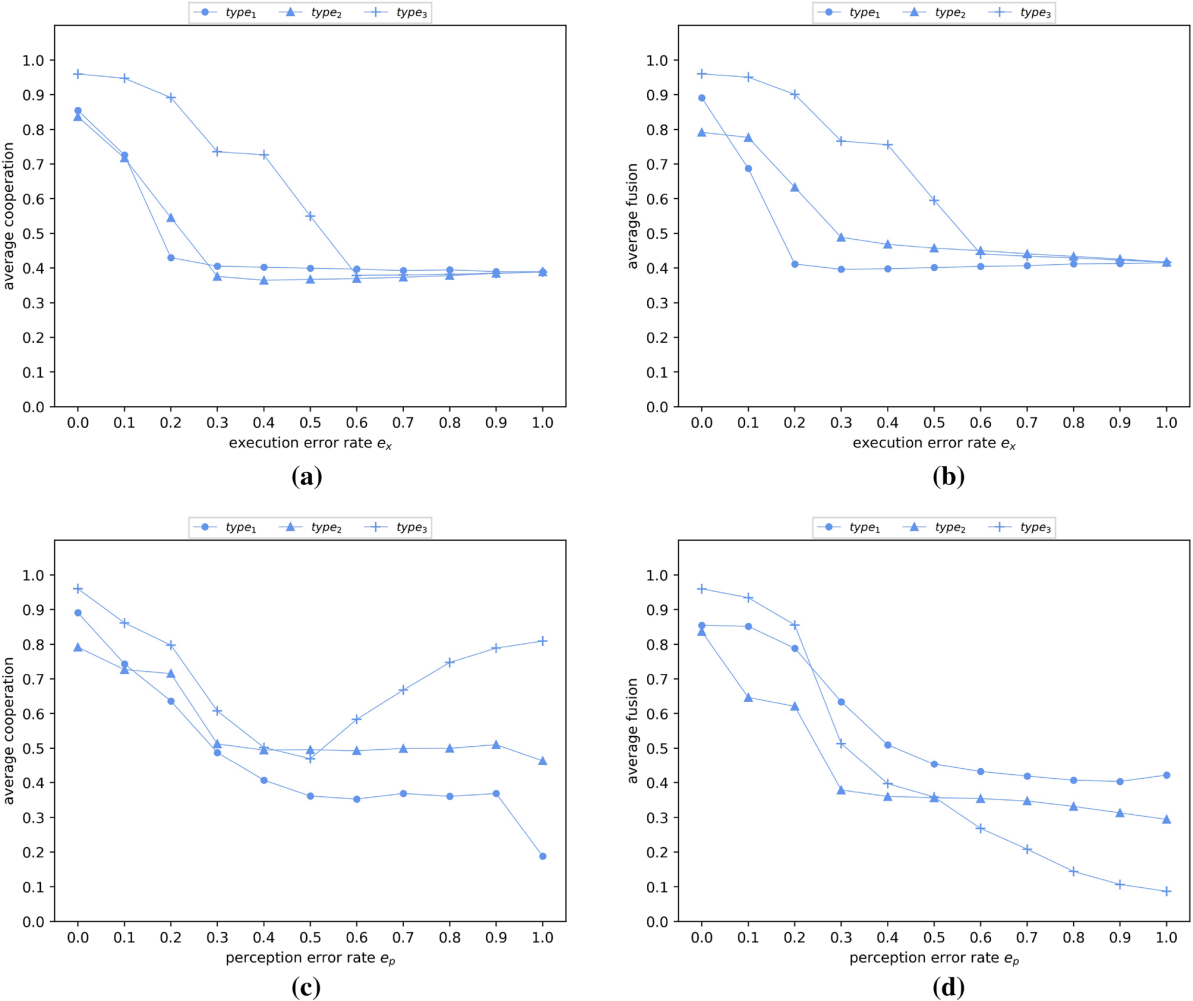
Average cooperation (**a**) and fusion (**b**) as a function of the rate of execution error $e_x$. Average cooperation (**c**) and fusion (**d**) as a function of the rate of perception error $e_p$. Ostracism of type 1, 2, or 3 is performed by each agent *i* with a probability equal to the agent’s fusion level $f_i$. Perception error $e_p$ is defined as the probability of the individual’s contribution to the group being replaced by a random number in the range [0,1]. Execution error $e_x$ is defined as the probability for an agent not to perform ostracism when it should. Agents interact randomly with the other fused individuals within their in-group with a probability equal to their own fusion levels ($S_i = f_i$). The in-group of an agent *i* is defined as the subset of agents having at least same level of fusion as *i* (i.e., $f_i$ or greater). Agents interact randomly with the whole population with a probability equal to 1 − $S_i$. Results are averaged over five randomly seeded runs, each conducted for 50,000 generations. Parameter settings are consistent with Fig. 1.

To assess resilience, we consider the effect of errors upon an agent’s interpretation of exclusion in response to hypocrisy [Eq. (4)], and we assess the subsequent effects assuming type-1, type-2 and type-3 ostracism. Perception error corresponds to mis-information, where perception of an individual’s contribution to the group is replaced by a random number in the range [0, 1]. Execution error causes an agent to incorrectly respond to hypocrisy, by not performing ostracism when it should. These errors are governed by probabilities $e_p$ and $e_x$ respectively. Figure 5 shows that significantly different effects are caused by these alternative forms of error. However in both cases, at least 20% error can be tolerated under type-3 ostracism, while sustaining strong average cooperation and strong average fusion levels, reaching over 80% in both cases.

The more execution error $e_x$ is applied under any type of ostracism, the closer the model gets to the default of functioning without ostracism (type-0). Type-1 ostracism exhibits slightly more sensitivity to execution error (i.e., up to $e_x = 0.2$) after which the behaviour of type-1 and type-2 converge towards 0.4 for both average cooperation and average fusion. In combination, execution error in type-3 ostracism provides an additional layer of protection over execution error in type-1 ostracism, by ensuring that error in payoff accumulated through mis-execution has less chance of being compounded by ostracism at the reproduction step. This leads to robust performance: a 40% execution error rate under type-3 ostracism results in average cooperation and average fusion levels greater than 0.7.

In contrast to this, perception errors function by injecting noise in the signal on which the decision to perform ostracism is based, rather than using authentic information and failing to execute ostracism. As perception error increases, type-1 and type-2 ostracism result in average cooperation and average fusion behaving in a similar way, diminishing to a plateau in terms of average cooperation. However type-3 ostracism behaves significantly differently, which appears counter-intuitive on first sight. In particular, when perception error is more frequent ($e_p \ge 0.5$), average cooperation increases. However, this correlates with a decrease in fusion, and this signals that the compound effect of noise in ostracism at the interaction and selection stages combine to limit fusion taking hold. This in turn limits defective strategies from taking hold through reputation sharing and allows cooperative strategies to dominate, consistent with all agents having only a personal reputation and no fusion^53^. Note that these experiments are performed without errors being applied to the action rules, which govern the extent of cooperative behaviour.

### Identity fusion coevolves with cooperation in the presence of associated marginal increases in cost

While ostracism is regarded as a “cost-less punishment”^81^, those that invoke hypocrisy may incur an additional relative cost due to the overhead in collecting more information about other agents. Although this is a debatable issue (fused individuals may feel incentivised based on their shared identity with the group or may acquire information implicitly through cost-neutral social mechanisms such as gossiping), we consider a marginal cost for agents potentially instigating ostracism, as compared to those who function without (i.e., assuming type-0 ostracism).

**Figure 6:**
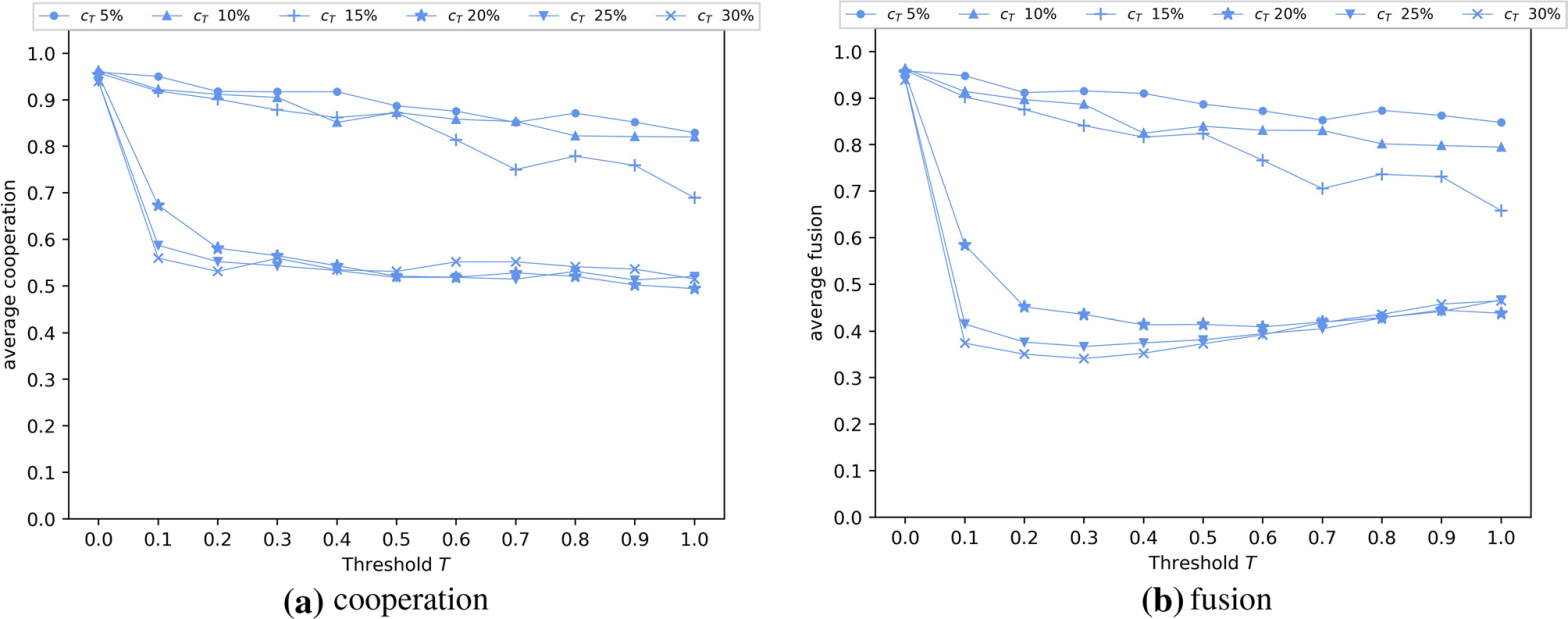
Average cooperation (**a**) and fusion (**b**) as a function of the cost threshold $T$ and for different rates of the additional cost $c_T$. Ostracism of type 3 is performed by each agent *i* with a probability equal to the agent’s fusion level $f_i$. Threshold $T$ defined as the minimum threshold that an agent’s fusion must reach ($f_i \ge T$) in order for ostracism to take place. Agents with a fusion level equal or below *T* incur a cost $c=0.7$ for a donation while agents with fusion levels above *T* incur a cost of $c+c_T$ for each donation. $c_T$ is defined in a range between $5\%$ and $30\%$ of *c*. Agents interact randomly with the other fused individuals within their in-group with a probability equal to their own fusion levels ($S_i = f_i$). The in-group of an agent *i* is defined as the subset of agents having at least same level of fusion as *i* (i.e., $f_i$ or greater). Agents interact randomly with the whole population with a probability equal to 1 − $S_i$. Results are averaged over five randomly seeded runs, each conducted for 50,000 generations. Parameter settings are consistent with Fig. 1.

Using threshold *T*, we introduce an additional cost $c_T$ for agents that invoke potential ostracism. Agents with fusion levels of at least *T* incur a cost of $c+c_T$ for each donation, where agents with a fusion level below *T* (i.e., don’t invoke potential ostracism) incur a cost *c* for a donation. Figure 6 shows the effect upon allowing $c_T$ to range between $5\%$ and $30\%$ of *c*, applying $c= 0.7$. The results show that the additional marginal cost has little impact on the evolution of cooperation and fusion, when $c_T$ is up to a level of around 15%.

Beyond $c_T = 0.15$, the additional cost represents a barrier to high fusion levels and cooperation. The disruptive effect of the differential cost on fusion and cooperation is interesting - note that when all agents incur a uniform cost $c+c_T$ for donation (i.e., $T=0$), average fusion and average cooperation is greater than when using the dual cost structure (i.e., cost $c+c_T$ only above threshold *T* where $T>0$). Agents are effectively trading a cost advantage against adopting a fusion level of *T* or beyond, and $c_T = 15\%$ is the approximate level at which additional costs impede this transition.

## Discussion

The results provide insight into how identity fusion is promoted alongside cooperation in support of a group. Consistent with the qualitative development of identity fusion^1^, an agent centric approach is modelled where the group is a passive entity that holds a reputation but no agency in is own right. Thus agents retain personal empowerment and have the autonomy to change their fusion level, which governs the extent to which an individual’s own identity is drawn from the group. This is represented by the fused agent’s personal embodiment of the group’s reputation. Natural selection is invoked to observe the evolution of agents’ fusion levels, alongside the agents strategy for cooperation, which is based on the social comparison of other agent’s reputation.

The saliency of fused agents’ personal identity is reflected through a form of in-group reputation, expressing their contribution towards the group. Vicarious hypocrisy is central to assessing this form of reputation between agents, the sensitivity to which heightens through identity fusion towards the group. This captures inconsistent behaviour from the perspective of a fused agent, concerning support for the group’s reputation given the extent of the agents’ fusion. When fusion is strong, vicarious dissonance indicates a threat to the individual agent, triggering cognitive dissonance and motivating a behavioural response to reconcile this effect - our results indicate that ostracism at both interaction and selection is preferential. Through this mechanism, the group implicitly benefits from the action of fused agents. This is also sustained when there is a modest cost differential associated with the ‘punishment overhead’ (up to around 15% additional cost). Consequently, fusion provides a coordination mechanism for collective action, where personal incentives drive a benefit that is shared across other fused individuals. A strong incentive for fused individuals to sustain their contribution towards the group is inherent, otherwise agents become vulnerable to being perceived as hypocrites themselves, resulting in ostracism diminishing their payoff and future selection opportunities. This drives the evolution of fusion, with fused agents effectively gaining an advantage from the support of fused others, against shirkers.

The coordination mechanism for ostracism, invoked through identity fusion towards a group, is particularly noteworthy. From the perspective of human cooperation, altruistic punishment of shirking behaviour in humans has been acknowledged as key in promoting indirect reciprocity^109^, particularly in the context of a group, which is central to the organisation and success of human cooperation. Such punishment represents collective action^110^, where individuals undertake a potentially costly individual action that benefits the wider group and introduces a second-order free rider problem. However it has remained a longstanding puzzle as to how such collective action arises^83^, with a variety of alternative mechanisms proposed without consensus. A significant number of proposals align with human psychological capabilities, such as an individual becoming culturally conditioned by social norms^111–113^, neural satisfaction from punishing norm violation^114,115^, egalitarianism^116^, morality^117^ or influence due to negative emotions^109^. However, the belief system that underpin these dispositions is likely to occur as the consequence of an individual’s identity, including their psychological interdependence with their in-group^118^. Noting that this can be expressed through identity fusion^119^, and based on our model that demonstrates how identity fusion can transfer group objectives to the individual, we hypothesise that identity fusion unifies the explanation of how collective action enables the resourcing and coordination of seemingly altruistic punishment.

An important point to note however, is that our findings are based on fusion evolving entirely from dyadic encounters rather than from multimodal encounters, which is arguably more realistic. Different dynamics may result under such conditions. Furthermore, perception of in-group violations in our model assume public knowledge. In practice this may well not be the case^120^ and its important to consider the consequences of identity fusion not being globally visible. Our results on execution and perception errors provide some initial insights in this direction, indicating that reasonable level of obfuscation can be sustained (e.g., 40% execution error rate under type-3 ostracism) while allowing fusion and cooperation to evolve. However this may change considerably when observations are to some degree private, and individual agents may be positioned to make different private observations. Investigation of such a scenario may benefit from using a social network to structure the agents, which also allows hetrogeneous relationships between individuals to come into play.

The evolution of identity fusion through our model also provides a new perspective on group or multi-level selection. This is a longstanding issue of considerable debate, where it is proposed that selection can act on groups as well as their constituent members. Pinker^121^ strongly argues that this is an illusion, with no plausible mechanism for a ‘group gene’ facilitating selection. It is argued that this illusion exists as a consequence of the power, influence and manipulation exercised by a group over its members (e.g., through monotheistic religions).

However, our model of reputation, based on identity fusion towards a group^7^, provides an explanation as to how group level selection may appear to be a phenomenon in its own right, while actually its the individuals that are fused with the group’s identity that give the basis for selection. Because identity fusion enables individuals to take a degree of the group identity as their own, and the group benefits (e.g., high reputation) are felt by the single individual, the group is providing utility and advantage that can be propagated to individuals in future generations. Our model demonstrates that this requires individuals to increasingly influence the contribution of others towards the group as they become increasingly fused, which is plausible because individuals become more dependent on the group’s reputation under these circumstances. Detecting hypocrisy is therefore individually incentivised, but the benefits spread to the group. In this way, fused individuals can gain an evolutionary advantage, that helps to explain at least partially, why a notion of group-level evolution may appear to present itself as a selection mechanism. This also highlights that hypocrisy, as an instinctive human state, may have been realised as a mechanism to provide advantage to those engaging in identity fusion.

Representing our model in a computational form has enabled observation of important dynamics that are sufficient to promote the evolution of identity fusion in digital agents, while also sustaining cooperation. These dynamics embody simple representations of human behaviour and give clues as to their possible ecological importance, indicating new directions for experimental validation in a human context. One such avenue is the role of hypocrisy with respect to group identity, which has not been central in the original conceptualisation of identity fusion but, we argue, represents a novel and straightforward response behaviour that is likely invoked through heightened fusion. As fused individuals are motivated to act in support of the group, it is plausible that they would also expect such behaviour in other group members, with heightened sensitivity to group members who, despite their group membership, do not act in the group’s interests. Such sensitivity to hypocrisy is finely tuned in humans and can be understood, in part, as a form of false signalling^122^. Furthermore, observers tend to be quite sensitive to violations of norms of in-group loyalty^123^, suggesting that such processes would be even more powerful in highly fused individuals. Thus, an empirical approach could assess the relationship between identity fusion and sensitivity to the behaviour of other individuals towards the group. For example, are highly fused individuals more sensitive to the group-related behaviour of others? Are they more likely to ostracise of otherwise impose costs on group members who fail to act in the group’s interests? Answering these questions via experimental research would be a valuable way to align model predictions with human behaviour. Such experiments would involve observing how participants, expressing a range of fusion levels towards a particular group, respond to the potentially hypocritical actions of others. Existing approaches^122,123^ provide useful experimental designs that could be further augmented to consider identity fusion, where data can be collected online or through in-lab participation.

Additionally, we note that how identity fusion affects choices for interaction between individuals is an interesting direction for empirical research that is stimulated by this work. For example, it would be useful to examine whether individual-level fusion increases the tendency to preferentially interact with other in-group members, as optionally considered in our model; while the tendency to prefer in-group partners in general has been previously observed^124^, this has not yet been explicitly linked to identity fusion per se, and would contribute further insights concerning homophily and identity. Finally, the model also highlights opportunities to explore how fused individuals might consider their reputation as contingent on the group’s reputation. Arguments on how groups are used to define one’s social self^125^ are relevant here, in particular how individuals perceive they are evaluated given the groups to which they belong. These questions would also be readily open to exploration through experimentation or observation.

## Methods

We consider the evolution of indirect reciprocity based on the donation game, a special case of the mutual aid game^83^ assuming a single donor. We extend the social comparison of reputation^53^ to consider the evolution of identity fusion of agents towards a group *G*. An agent’s identity fusion is modelled through its composition of reputation (see Reputation Section) and the extent to which it draws on the group’s reputation. Agents are randomly selected to play the donation game. Two components affect how this is conducted - firstly the agent’s action rules (see Action Rules Section), and secondly the agent’s fusion level, which may lead the agent to considering their in-group standing and the potential hypocrisy of the other player (see Section on Personal Identity of Fused Agents and Hypocrisy). The donor’s reputation is updated (see Assessment Rules Section). After 5000 donation games, which constitutes one generation, natural selection is performed (see Selection and Reproduction Section). This cycle is repeated for 50,000 generations. Initial action rules and fusion levels are randomly assigned and results represent the average of 5 randomly seeded runs. Key variables are summarised in Figure 1.

### Reputation

An agent *i*’s fusion level $f_i$ towards group *G* determines the extent that its reputation $r^i$ is composed of the group’s reputation $r_G$. Specifically *i*’s integrated reputation is $r^i = (1-f_i)r_i + f_ir_G$ where $r_i$ indicates the agent *i*’s personal reputation, consistent with the Venn diagram overlap approach through which human subjects express the extent of their identity fusion towards a group^54^. $r_i$ and $r_G$ are represented as integers and are capped in the range [-5,5]^53^. However, whether or not agent *i* has non-zero fusion towards *G* affects how *i* views *j*’s reputation. When $f_i = 0$, we assume that *i* is not positioned to value the group reputation $r_G$ and therefore *i* views only *j*’s isolated personal reputation (i.e., *i* assumes $r^j = r_j$) irrespective of *j*’s fusion level. When $f_i > 0$, then *i* views *j*’s reputation taking into account the extent of *j*’s fusion level (i.e., *i* assumes $r^j = (1-f_j)r_j + f_jr_G$).

### Personal identity of fused agents and hypocrisy

Within each generation, an agent’s contribution to supporting group *G* is defined as $ctr_{i} = \frac{n_i^{pos} + n_i^{neu}}{n_i^{pos} + n_i^{neu} + n_i^{neg}}$, where: $n_i^{pos}$ is the number of actions made by *i* that result in an increase to $r_G$; $n_i^{neu}$ is the number of actions made by *i* (legitimate defections) that don’t invoke a change to $r_G$; $n_i^{neg}$ is the number of actions made by *i* that reduce $r_G$. $ctr_i$ is set to zero at the beginning of each generation. $ctr_i$ represents an additional form of personal reputation for fused agents through which their individuality can be assessed. A fused agent *i* ($f_i>0$) views another fused agent *j* ($f_j > 0$) as a hypocrite if and only if both $f_j \ge f_i \text{ and } ctr_j < \ ctr_i$.

Hypocrite detection is used by agent *i* to determine whether or not a form of ostracism is performed against *j*. We also invoke a minimum threshold *T*, $(0< T < 1)$ that an agent’s fusion level must reach for any ostracism to take place. When $f_i \ge T$, then either *type-1, type-2* or *type-3* ostracism is invoked with probability $p=f_i$. Under *type-1* ostracism, *i* ignores its action rules (see Action Rule Section) and does not undertake a donation towards *j*. This may result in a penalty for its personal reputation $r_i$. Under *type-2* ostracism, *i* excludes *j* at reproduction (see Reproduction Section). Under *type-3* ostracism *i* invokes both type-1 and type-2 ostracism. *type-0* is used to indicate that no ostracism is undertaken.

### Action rules

In each generation we perform 5000 random agent selections where each selected agent *i* plays the donation game. The probability that *i* plays against a randomly selected member of *i*’s in-group (i.e., *i* and *j* have at least $f_i$ fusion in common) is $S_i$. Either $S_i$ is exogenously fixed or $S_i = f_i$. When $S_i = 0$ *j* is randomly selected from the whole population. If type-1 ostracism is invoked and *i* ostracises *j*, then *i* makes no donation to *j* irrespective of its action rules. Otherwise *i* considers its action rules, as defined by the binary variables $u_i$, $d_i$ and $s_i$. These determine whether or not *i* donates when similarity $(s_i)$, upward comparison $(u_i)$ or downward comparison $(d_i)$ is observed by *i* in respect of the potential recipient *j*’s reputation ($r^j$), as compared to *i*’s reputation ($r^i$). Similarity occurs when $r^j-\Delta \le r^i \le r^j+\Delta$, upward self-comparison occurs when $r^j>r^i + \Delta$, and downward self-comparison occurs when $r^j<r^i - \Delta$. We set $\Delta = 0$. If *i* donates to *j* then *i* incurs a cost $c+c_T$ and *j* receives a benefit *b*, and we apply a cost to benefit ratio of $c = 0.7$ and $b=1$. $c_T$ represents a potential additional cost due to detecting hypocrisy, and is set as $c_T = 0$ unless otherwise stated. An agent’s payoff for a particular generation represents the total payoff less total costs that are incurred from participation in donation games. Payoff is set to zero at the beginning of each generation.

### Assessment

Personal ($r_i$) and group ($r_G$) reputations are updated in response to an agent’s donation behaviour, based on the whether *i* is fused. The principle of standing^88,93,94^ is applied. If *i* donates to *j* then both $r_i$ and $r_G$ are incremented. If *i* defects on *j* and *j* is at least as reputable as *i* ($r^j \ge r^i$) then both $r_i$ and $r_G$ are decremented. If *i* defects on *j* and *j* is less reputable than *i* ($r^j < r^i$) both $r_i$ and $r_G$ remain unchanged. These rules assume that *i* is fused ($f_i>0$) and is therefore dependent on both $r_i$ and $r_G$. When $f_i=0$ the above updating rules are only applied to $r_i$. These updating rules apply in response to donation decisions made using the action rules. If *i* defects on *j* due to type-1 ostracism, then $r_i$ is decremented if and only if $r^j\ge r^i$ and no penalty to $r_G$ occurs since from the perspective of *G*, the defection is legitimate.

### Selection and reproduction

At the end of each generation we apply clonal reproduction, which is dependent on a single parent being replaced by an agent selected from the current population. This is commonly used in previous studies on the evolution of indirect reciprocity^42,44,96,97^. Payoff is applied as the fitness function, and if type-2 ostracism is invoked then an agent *i* excludes from consideration any agent *j* that it views as hypocritical (see Hypocrisy Section). When an agent *k* is selected, its action rule and fusion level is copied and carried forward to create an offspring agent in the new generation. This process is repeated for all agents *i* in the current generation. For each agent *k* in the new generation, $s_k, u_k, d_k$ and $f_k$ are each randomly mutated at the rate of $\mu =1/100$. Payoff and all reputation variables are set to zero in preparation for the next generation to commence.

### Perception and execution errors

Perception error ($e_p$) corresponds to mis-information, where perception of an individual’s contribution ($ctr_j$) to the group is replaced by a random number in the range [0, 1]. Execution error ($e_x$) causes an agent to incorrectly respond to hypocrisy, by not performing ostracism when it should, as defined in the Hypocrisy Section above. These can be to applied to type-1, type-2 and type-3 ostracism. Results indicate when these are optionally applied.
